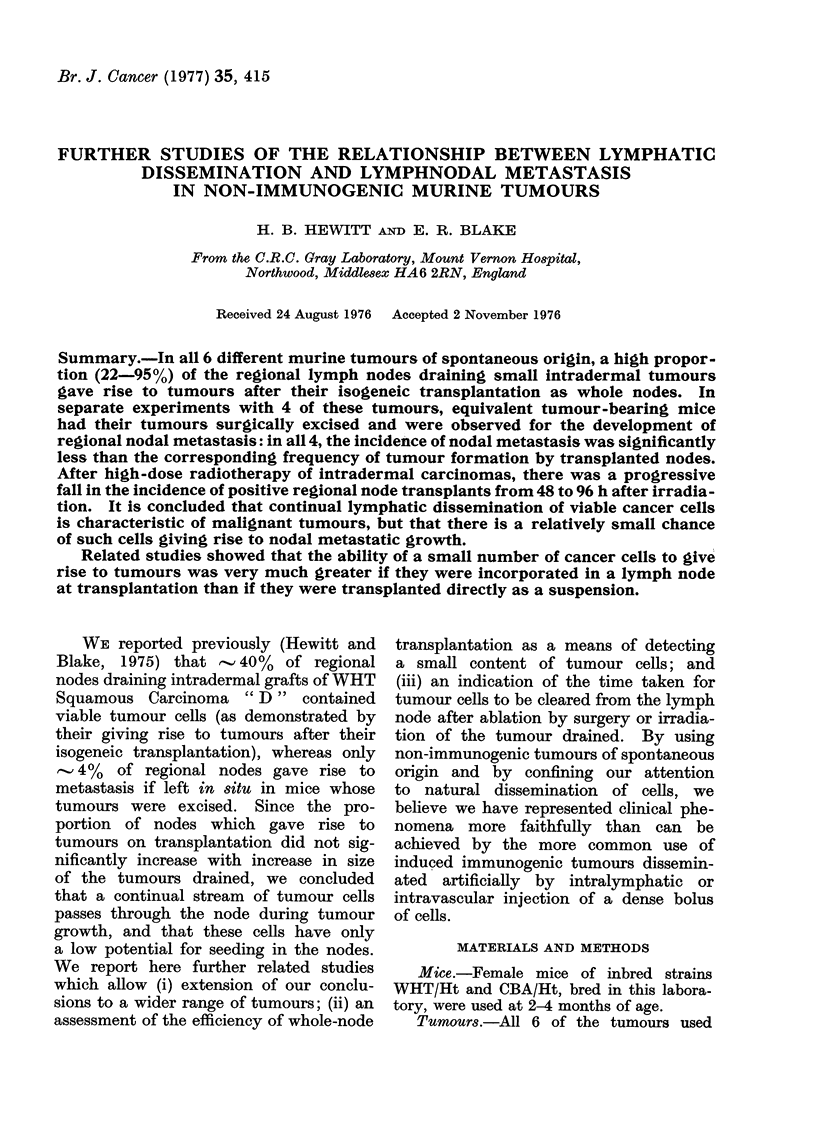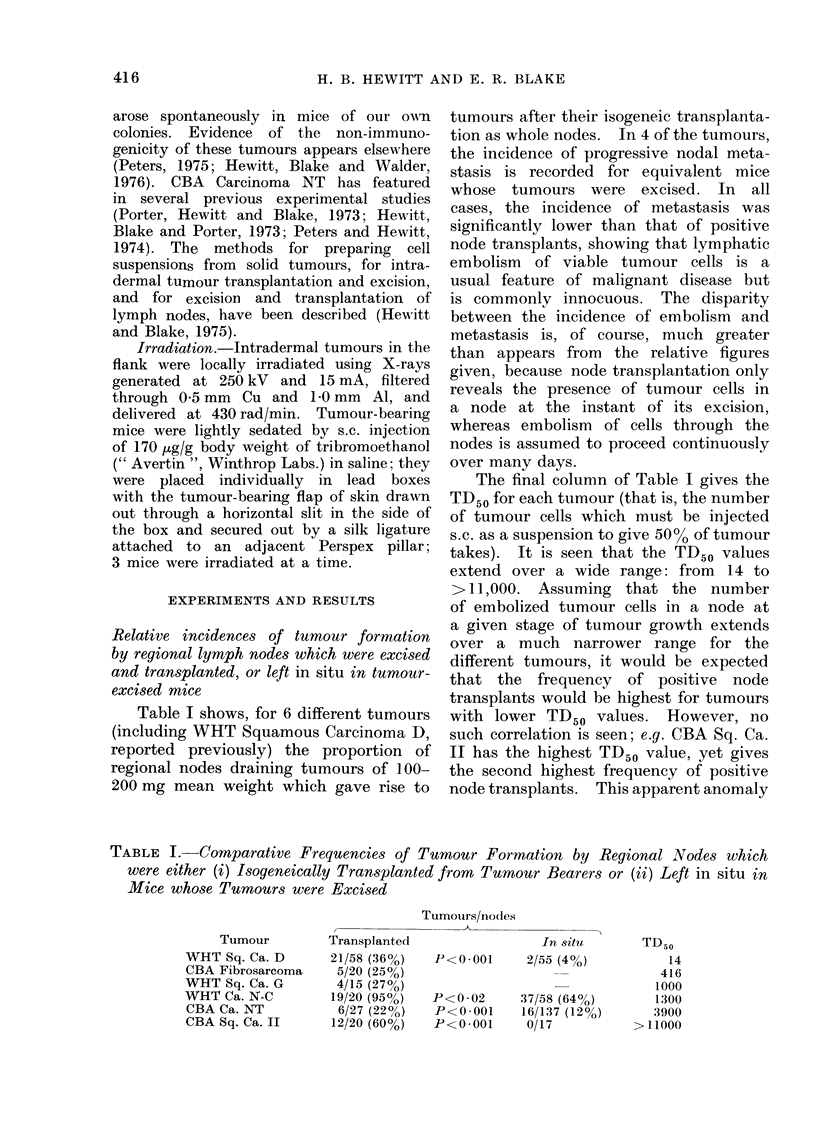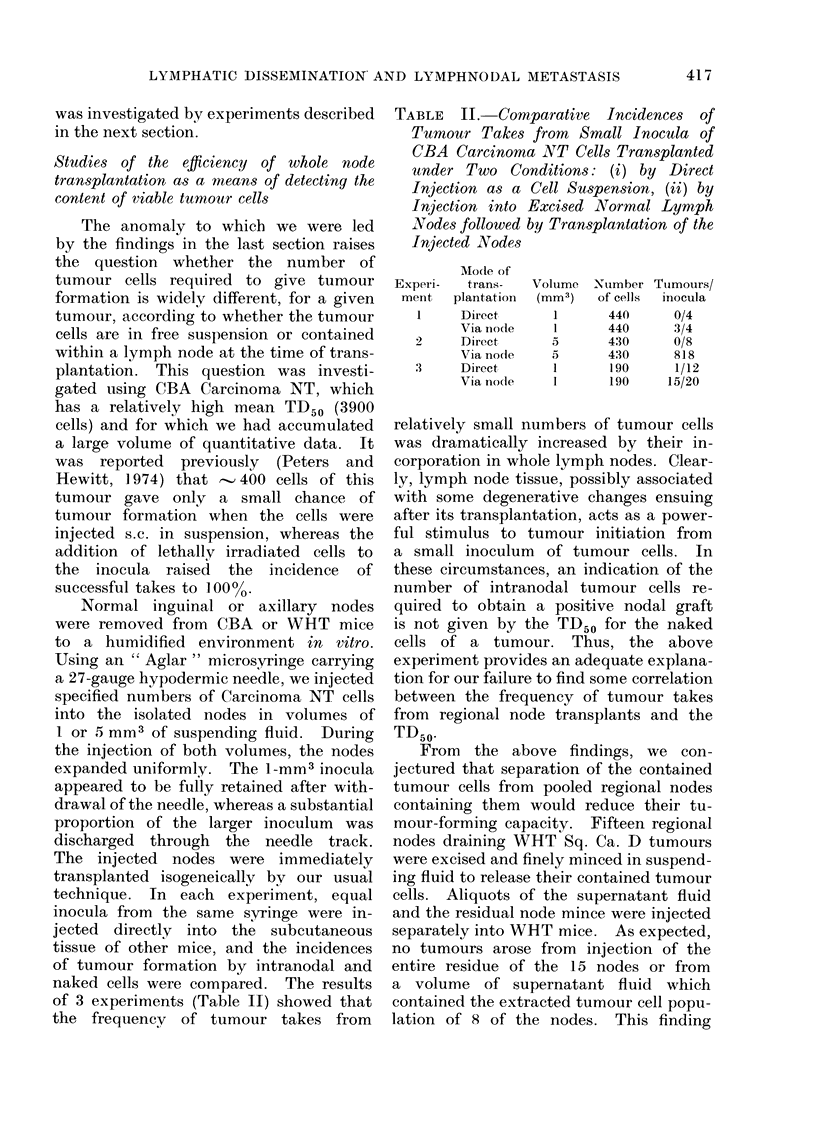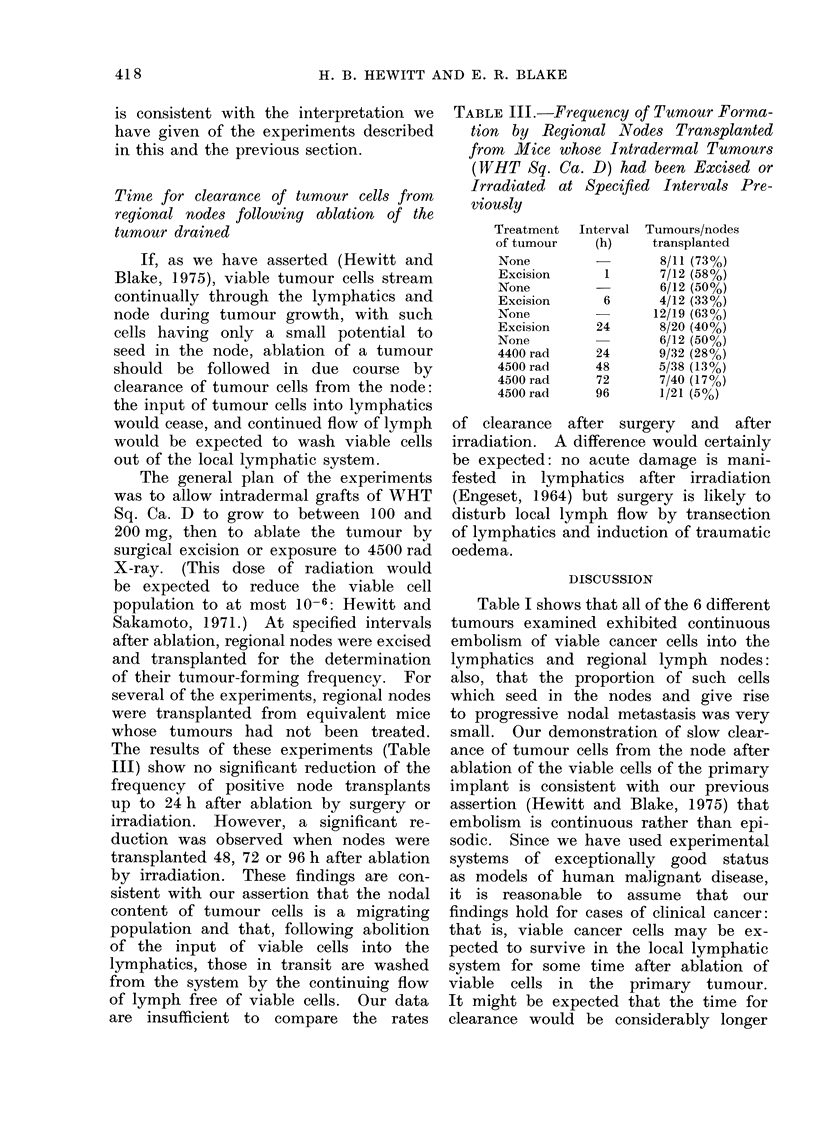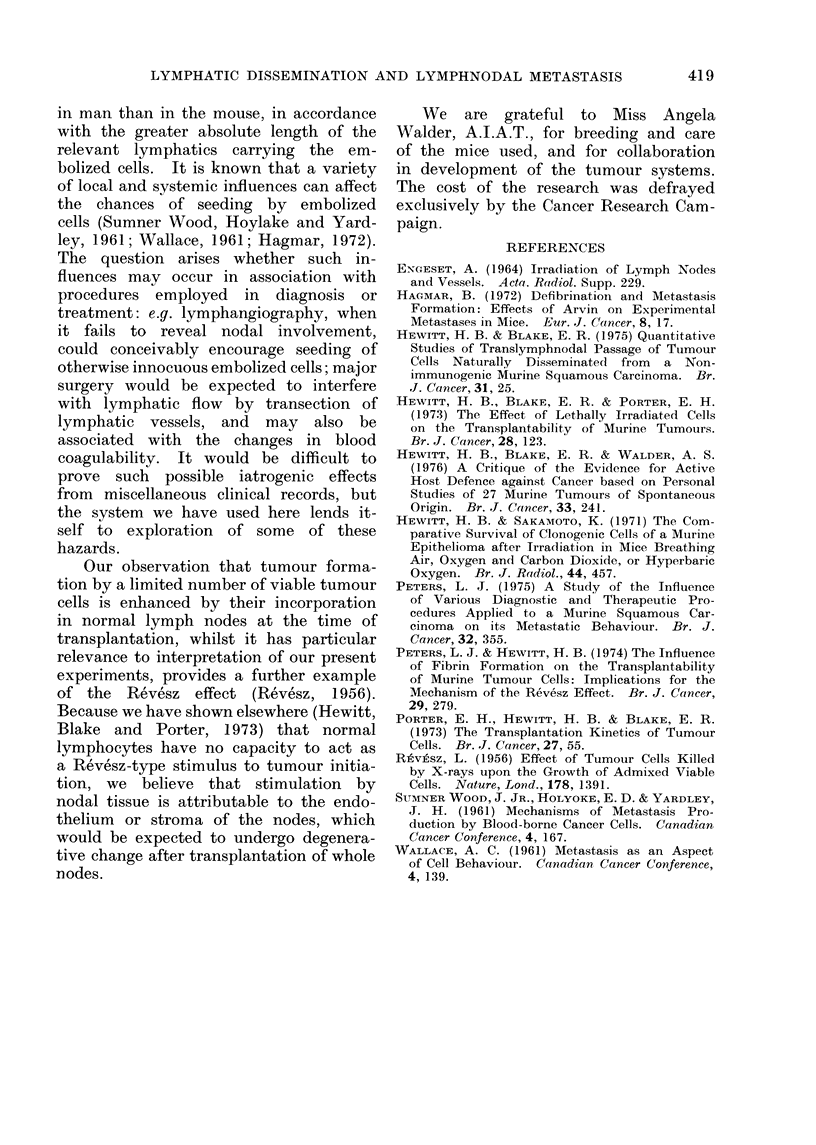# Further studies of the relationship between lymphatic dissemination and lymphnodal metastasis in non-immunogenic murine tumours.

**DOI:** 10.1038/bjc.1977.62

**Published:** 1977-04

**Authors:** H. B. Hewitt, E. R. Blake

## Abstract

In all 6 different murine tumours of spontaneous origin, a high proportion (22-95%) of the regional lympgh nodes draining small intradermal tumours gave rise to tumours after their isogeneic transplantation as whole nodes. In separate experiments with 4 of these tumours, equivalent tumour-bearing mice had their tumours surgically excised and were observed for the development of regional nodal corresponding frequency of tumour formation by transplanted nodes. After high-dose radiotherapy of intradermal carcinomas, there was a progressive fall in the incidence of positive regional node transplants from 48 to 96 h after irradiation. It is concluded that continual lymphatic dissemination of viable cancer cells is characteristic of malignant tumours, but that there is a relatively small chance of such cells giving rise to nodal metastatic growth. Related studies showed that the ability of a small number of cancer cells to give rise to tumours was very much greater if they were incorporated in a lymph node at transplantation than if they were transplanted directly as a suspension.


*Br. J. Cancer* (1977) **35**, 415

### FURTHER STUDIES OF THE RELATIONSHIP BETWEEN LYMPHATIC DISSEMINATION AND LYMPHNODAL METASTASIS IN NON-IMMUNOGENIC MURINE TUMOURS

H. B. HEWITT AND E. R. BLAKE

*From the C.R.C. Gray Laboratory, Mount Vernon Hospital, Northwood, Middlesex HA6 2RN, England*

Received 24 August 1976 Accepted 2 November 1976

**Summary.—In all 6 different murine tumours of spontaneous origin, a high proportion (22—95%) of the regional lymph nodes draining small intradermal tumours gave rise to tumours after their isogeneic transplantation as whole nodes. In separate experiments with 4 of these tumours, equivalent tumour-bearing mice had their tumours surgically excised and were observed for the development of regional nodal metastasis: in all 4, the incidence of nodal metastasis was significantly less than the corresponding frequency of tumour formation by transplanted nodes. After high-dose radiotherapy of intradermal carcinomas, there was a progressive fall in the incidence of positive regional node transplants from 48 to 96 h after irradiation. It is concluded that continual lymphatic dissemination of viable cancer cells is characteristic of malignant tumours, but that there is a relatively small chance of such cells giving rise to nodal metastatic growth.**

**Related studies showed that the ability of a small number of cancer cells to give rise to tumours was very much greater if they were incorporated in a lymph node at transplantation than if they were transplanted directly as a suspension.**

WE reported previously (Hewitt and Blake, 1975) that $\sim 40\%$ of regional nodes draining intradermal grafts of WHT Squamous Carcinoma "D" contained viable tumour cells (as demonstrated by their giving rise to tumours after their isogeneic transplantation), whereas only $\sim 4\%$ of regional nodes gave rise to metastasis if left *in situ* in mice whose tumours were excised. Since the proportion of nodes which gave rise to tumours on transplantation did not significantly increase with increase in size of the tumours drained, we concluded that a continual stream of tumour cells passes through the node during tumour growth, and that these cells have only a low potential for seeding in the nodes. We report here further related studies which allow (i) extension of our conclusions to a wider range of tumours; (ii) an assessment of the efficiency of whole-node transplantation as a means of detecting a small content of tumour cells; and (iii) an indication of the time taken for tumour cells to be cleared from the lymph node after ablation by surgery or irradiation of the tumour drained. By using non-immunogenic tumours of spontaneous origin and by confining our attention to natural dissemination of cells, we believe we have represented clinical phenomena more faithfully than can be achieved by the more common use of induced immunogenic tumours disseminated artificially by intralymphatic or intravascular injection of a dense bolus of cells.

### MATERIALS AND METHODS

*Mice.*—Female mice of inbred strains WHT/Ht and CBA/Ht, bred in this laboratory, were used at 2–4 months of age.

*Tumours.*—All 6 of the tumours used arose spontaneously in mice of our own colonies. Evidence of the non-immunogenicity of these tumours appears elsewhere (Peters, 1975; Hewitt, Blake and Walder, 1976). CBA Carcinoma NT has featured in several previous experimental studies (Porter, Hewitt and Blake, 1973; Hewitt, Blake and Porter, 1973; Peters and Hewitt, 1974). The methods for preparing cell suspensions from solid tumours, for intradermal tumour transplantation and excision, and for excision and transplantation of lymph nodes, have been described (Hewitt and Blake, 1975).

*Irradiation*.—Intradermal tumours in the flank were locally irradiated using X-rays generated at 250 kV and 15 mA, filtered through 0·5 mm Cu and 1·0 mm Al, and delivered at 430 rad/min. Tumour-bearing mice were lightly sedated by s.c. injection of 170 $\mu$g/g body weight of tribromoethanol (" Avertin ", Winthrop Labs.) in saline; they were placed individually in lead boxes with the tumour-bearing flap of skin drawn out through a horizontal slit in the side of the box and secured out by a silk ligature attached to an adjacent Perspex pillar; 3 mice were irradiated at a time.

### EXPERIMENTS AND RESULTS

*Relative incidences of tumour formation by regional lymph nodes which were excised and transplanted, or left* in situ *in tumour-excised mice*

Table I shows, for 6 different tumours (including WHT Squamous Carcinoma D, reported previously) the proportion of regional nodes draining tumours of 100–200 mg mean weight which gave rise to tumours after their isogeneic transplantation as whole nodes. In 4 of the tumours, the incidence of progressive nodal metastasis is recorded for equivalent mice whose tumours were excised. In all cases, the incidence of metastasis was significantly lower than that of positive node transplants, showing that lymphatic embolism of viable tumour cells is a usual feature of malignant disease but is commonly innocuous. The disparity between the incidence of embolism and metastasis is, of course, much greater than appears from the relative figures given, because node transplantation only reveals the presence of tumour cells in a node at the instant of its excision, whereas embolism of cells through the nodes is assumed to proceed continuously over many days.

The final column of Table I gives the $TD_{50}$ for each tumour (that is, the number of tumour cells which must be injected s.c. as a suspension to give 50% of tumour takes). It is seen that the $TD_{50}$ values extend over a wide range: from 14 to >11,000. Assuming that the number of embolized tumour cells in a node at a given stage of tumour growth extends over a much narrower range for the different tumours, it would be expected that the frequency of positive node transplants would be highest for tumours with lower $TD_{50}$ values. However, no such correlation is seen; *e.g.* CBA Sq. Ca. II has the highest $TD_{50}$ value, yet gives the second highest frequency of positive node transplants. This apparent anomaly

TABLE I.—*Comparative Frequencies of Tumour Formation by Regional Nodes which were either (i) Isogeneically Transplanted from Tumour Bearers or (ii) Left* in situ *in Mice whose Tumours were Excised*

| Tumour | Tumours/nodes: Transplanted | | Tumours/nodes: *In situ* | $TD_{50}$ |
|---|---|---|---|---|
| WHT Sq. Ca. D | 21/58 (36%) | $P<0·001$ | 2/55 (4%) | 14 |
| CBA Fibrosarcoma | 5/20 (25%) | | — | 416 |
| WHT Sq. Ca. G | 4/15 (27%) | | — | 1000 |
| WHT Ca. N-C | 19/20 (95%) | $P<0·02$ | 37/58 (64%) | 1300 |
| CBA Ca. NT | 6/27 (22%) | $P<0·001$ | 16/137 (12%) | 3900 |
| CBA Sq. Ca. II | 12/20 (60%) | $P<0·001$ | 0/17 | >11000 |

was investigated by experiments described in the next section.

*Studies of the efficiency of whole node transplantation as a means of detecting the content of viable tumour cells*

The anomaly to which we were led by the findings in the last section raises the question whether the number of tumour cells required to give tumour formation is widely different, for a given tumour, according to whether the tumour cells are in free suspension or contained within a lymph node at the time of transplantation. This question was investigated using CBA Carcinoma NT, which has a relatively high mean $TD_{50}$ (3900 cells) and for which we had accumulated a large volume of quantitative data. It was reported previously (Peters and Hewitt, 1974) that ~400 cells of this tumour gave only a small chance of tumour formation when the cells were injected s.c. in suspension, whereas the addition of lethally irradiated cells to the inocula raised the incidence of successful takes to 100%.

Normal inguinal or axillary nodes were removed from CBA or WHT mice to a humidified environment *in vitro*. Using an "Aglar" microsyringe carrying a 27-gauge hypodermic needle, we injected specified numbers of Carcinoma NT cells into the isolated nodes in volumes of 1 or 5 $mm^3$ of suspending fluid. During the injection of both volumes, the nodes expanded uniformly. The 1-$mm^3$ inocula appeared to be fully retained after withdrawal of the needle, whereas a substantial proportion of the larger inoculum was discharged through the needle track. The injected nodes were immediately transplanted isogeneically by our usual technique. In each experiment, equal inocula from the same syringe were injected directly into the subcutaneous tissue of other mice, and the incidences of tumour formation by intranodal and naked cells were compared. The results of 3 experiments (Table II) showed that the frequency of tumour takes from

TABLE II.—*Comparative Incidences of Tumour Takes from Small Inocula of CBA Carcinoma NT Cells Transplanted under Two Conditions: (i) by Direct Injection as a Cell Suspension, (ii) by Injection into Excised Normal Lymph Nodes followed by Transplantation of the Injected Nodes*

| Experiment | Mode of transplantation | Volume ($mm^3$) | Number of cells | Tumours/inocula |
|---|---|---|---|---|
| 1 | Direct | 1 | 440 | 0/4 |
| | Via node | 1 | 440 | 3/4 |
| 2 | Direct | 5 | 430 | 0/8 |
| | Via node | 5 | 430 | 818 |
| 3 | Direct | 1 | 190 | 1/12 |
| | Via node | 1 | 190 | 15/20 |

relatively small numbers of tumour cells was dramatically increased by their incorporation in whole lymph nodes. Clearly, lymph node tissue, possibly associated with some degenerative changes ensuing after its transplantation, acts as a powerful stimulus to tumour initiation from a small inoculum of tumour cells. In these circumstances, an indication of the number of intranodal tumour cells required to obtain a positive nodal graft is not given by the $TD_{50}$ for the naked cells of a tumour. Thus, the above experiment provides an adequate explanation for our failure to find some correlation between the frequency of tumour takes from regional node transplants and the $TD_{50}$.

From the above findings, we conjectured that separation of the contained tumour cells from pooled regional nodes containing them would reduce their tumour-forming capacity. Fifteen regional nodes draining WHT Sq. Ca. D tumours were excised and finely minced in suspending fluid to release their contained tumour cells. Aliquots of the supernatant fluid and the residual node mince were injected separately into WHT mice. As expected, no tumours arose from injection of the entire residue of the 15 nodes or from a volume of supernatant fluid which contained the extracted tumour cell population of 8 of the nodes. This finding is consistent with the interpretation we have given of the experiments described in this and the previous section.

*Time for clearance of tumour cells from regional nodes following ablation of the tumour drained*

If, as we have asserted (Hewitt and Blake, 1975), viable tumour cells stream continually through the lymphatics and node during tumour growth, with such cells having only a small potential to seed in the node, ablation of a tumour should be followed in due course by clearance of tumour cells from the node: the input of tumour cells into lymphatics would cease, and continued flow of lymph would be expected to wash viable cells out of the local lymphatic system.

The general plan of the experiments was to allow intradermal grafts of WHT Sq. Ca. D to grow to between 100 and 200 mg, then to ablate the tumour by surgical excision or exposure to 4500 rad X-ray. (This dose of radiation would be expected to reduce the viable cell population to at most $10^{-6}$: Hewitt and Sakamoto, 1971.) At specified intervals after ablation, regional nodes were excised and transplanted for the determination of their tumour-forming frequency. For several of the experiments, regional nodes were transplanted from equivalent mice whose tumours had not been treated. The results of these experiments (Table III) show no significant reduction of the frequency of positive node transplants up to 24 h after ablation by surgery or irradiation. However, a significant reduction was observed when nodes were transplanted 48, 72 or 96 h after ablation by irradiation. These findings are consistent with our assertion that the nodal content of tumour cells is a migrating population and that, following abolition of the input of viable cells into the lymphatics, those in transit are washed from the system by the continuing flow of lymph free of viable cells. Our data are insufficient to compare the rates of clearance after surgery and after irradiation. A difference would certainly be expected: no acute damage is manifested in lymphatics after irradiation (Engeset, 1964) but surgery is likely to disturb local lymph flow by transection of lymphatics and induction of traumatic oedema.

TABLE III.—*Frequency of Tumour Formation by Regional Nodes Transplanted from Mice whose Intradermal Tumours (WHT Sq. Ca. D) had been Excised or Irradiated at Specified Intervals Previously*

| Treatment of tumour | Interval (h) | Tumours/nodes transplanted |
|---|---|---|
| None | — | 8/11 (73%) |
| Excision | 1 | 7/12 (58%) |
| None | — | 6/12 (50%) |
| Excision | 6 | 4/12 (33%) |
| None | — | 12/19 (63%) |
| Excision | 24 | 8/20 (40%) |
| None | — | 6/12 (50%) |
| 4400 rad | 24 | 9/32 (28%) |
| 4500 rad | 48 | 5/38 (13%) |
| 4500 rad | 72 | 7/40 (17%) |
| 4500 rad | 96 | 1/21 (5%) |

### DISCUSSION

Table I shows that all of the 6 different tumours examined exhibited continuous embolism of viable cancer cells into the lymphatics and regional lymph nodes: also, that the proportion of such cells which seed in the nodes and give rise to progressive nodal metastasis was very small. Our demonstration of slow clearance of tumour cells from the node after ablation of the viable cells of the primary implant is consistent with our previous assertion (Hewitt and Blake, 1975) that embolism is continuous rather than episodic. Since we have used experimental systems of exceptionally good status as models of human malignant disease, it is reasonable to assume that our findings hold for cases of clinical cancer: that is, viable cancer cells may be expected to survive in the local lymphatic system for some time after ablation of viable cells in the primary tumour. It might be expected that the time for clearance would be considerably longer in man than in the mouse, in accordance with the greater absolute length of the relevant lymphatics carrying the embolized cells. It is known that a variety of local and systemic influences can affect the chances of seeding by embolized cells (Sumner Wood, Hoylake and Yardley, 1961; Wallace, 1961; Hagmar, 1972). The question arises whether such influences may occur in association with procedures employed in diagnosis or treatment: *e.g.* lymphangiography, when it fails to reveal nodal involvement, could conceivably encourage seeding of otherwise innocuous embolized cells; major surgery would be expected to interfere with lymphatic flow by transection of lymphatic vessels, and may also be associated with the changes in blood coagulability. It would be difficult to prove such possible iatrogenic effects from miscellaneous clinical records, but the system we have used here lends itself to exploration of some of these hazards.

Our observation that tumour formation by a limited number of viable tumour cells is enhanced by their incorporation in normal lymph nodes at the time of transplantation, whilst it has particular relevance to interpretation of our present experiments, provides a further example of the Révész effect (Révész, 1956). Because we have shown elsewhere (Hewitt, Blake and Porter, 1973) that normal lymphocytes have no capacity to act as a Révész-type stimulus to tumour initiation, we believe that stimulation by nodal tissue is attributable to the endothelium or stroma of the nodes, which would be expected to undergo degenerative change after transplantation of whole nodes.

We are grateful to Miss Angela Walder, A.I.A.T., for breeding and care of the mice used, and for collaboration in development of the tumour systems. The cost of the research was defrayed exclusively by the Cancer Research Campaign.

### REFERENCES

ENGESET, A. (1964) Irradiation of Lymph Nodes and Vessels. *Acta. Radiol.* Supp. 229.

HAGMAR, B. (1972) Defibrination and Metastasis Formation: Effects of Arvin on Experimental Metastases in Mice. *Eur. J. Cancer*, **8**, 17.

HEWITT, H. B. & BLAKE, E. R. (1975) Quantitative Studies of Translymphnodal Passage of Tumour Cells Naturally Disseminated from a Non-immunogenic Murine Squamous Carcinoma. *Br. J. Cancer*, **31**, 25.

HEWITT, H. B., BLAKE, E. R. & PORTER, E. H. (1973) The Effect of Lethally Irradiated Cells on the Transplantability of Murine Tumours. *Br. J. Cancer*, **28**, 123.

HEWITT, H. B., BLAKE, E. R. & WALDER, A. S. (1976) A Critique of the Evidence for Active Host Defence against Cancer based on Personal Studies of 27 Murine Tumours of Spontaneous Origin. *Br. J. Cancer*, **33**, 241.

HEWITT, H. B. & SAKAMOTO, K. (1971) The Comparative Survival of Clonogenic Cells of a Murine Epithelioma after Irradiation in Mice Breathing Air, Oxygen and Carbon Dioxide, or Hyperbaric Oxygen. *Br. J. Radiol.*, **44**, 457.

PETERS, L. J. (1975) A Study of the Influence of Various Diagnostic and Therapeutic Procedures Applied to a Murine Squamous Carcinoma on its Metastatic Behaviour. *Br. J. Cancer*, **32**, 355.

PETERS, L. J. & HEWITT, H. B. (1974) The Influence of Fibrin Formation on the Transplantability of Murine Tumour Cells: Implications for the Mechanism of the Révész Effect. *Br. J. Cancer*, **29**, 279.

PORTER, E. H., HEWITT, H. B. & BLAKE, E. R. (1973) The Transplantation Kinetics of Tumour Cells. *Br. J. Cancer*, **27**, 55.

RÉVÉSZ, L. (1956) Effect of Tumour Cells Killed by X-rays upon the Growth of Admixed Viable Cells. *Nature, Lond.*, **178**, 1391.

SUMNER WOOD, J. JR., HOLYOKE, E. D. & YARDLEY, J. H. (1961) Mechanisms of Metastasis Production by Blood-borne Cancer Cells. *Canadian Cancer Conference*, **4**, 167.

WALLACE, A. C. (1961) Metastasis as an Aspect of Cell Behaviour. *Canadian Cancer Conference*, **4**, 139.